# Integrating clinical and research training in child psychiatry: fifteen-year outcomes of a federally supported program

**DOI:** 10.1186/s13034-020-00328-4

**Published:** 2020-05-14

**Authors:** Amanda Calhoun, Michael H. Bloch, Dorothy Stubbe, James F. Leckman, Andrés Martin

**Affiliations:** The Child Study Center, Yale School of Medicine, New Haven, CT USA

**Keywords:** Research training, Clinical training, Long-term outcomes, Child and adolescent psychiatry, Scientific independence

## Abstract

**Background:**

The Albert J. Solnit Integrated Training Program (AJSP) is an educational initiative designed to prepare physician-scientists for independent careers in the investigation and treatment of childhood psychiatric disorders.

**Methods:**

We compared fifteen cohorts (each representing a consecutive year of matriculation) of AJSP trainees and graduates (n = 30) to peers who were comparably ranked in our original match lists but ultimately pursued residency programs elsewhere (n = 60). Outcomes of interest between the two groups included professional affiliation, as measured by: (1) membership in the American Academy of Child and Adolescent Psychiatry (AACAP); and (2) certification by the American Board of Psychiatry and Neurology (ABPN), as well as three domains of research productivity: (1) Competitive awards received from AACAP; (2) Publication-related metrics derived from the National Library of Medicine (NLM); and (3) Federal grant funding from the National Institutes of Health (NIH).

**Results:**

AJSP participants were more commonly affiliated with AACAP and board certified in CAP. AJSP graduates and trainees outperformed their control group peers in several research outcomes: (1) Receipt of AACAP awards and number of awards per recipient were higher, and time to first award shorter in the AJSP than in the control group; (2) AJSP participants had more publications in PubMed, more first-authored publications, a higher h-index, and a shorter time to first publication than participants in the control group; and (3) NIH K- or R-series funding success rate was higher among AJSP participants (p < 0.05 for all comparisons).

**Conclusions:**

A program designed to support the development of clinician-scientists specifically dedicated to childhood mental health needs has been successful in fostering scientific creativity, productivity and independence. The expansion and replication of similar training initiatives will be an in important step forward to address the high level of morbidity and mortality associated with child and adolescent psychiatric disorders.

*We must shift our emphasis away from shepherding our trainees from one requirement or rotation safely onto the next. If we are to deliver on the latent promise of our discipline, we must train a new generation capable of redefining it. Our educational mission should be less about guiding and monitoring our pilgrims’ progress than about aggressively promoting our pipeline’s promise [* 1*].*

## Background

Concerns about the declining numbers of physician-scientists have existed for several decades. The ‘clinical investigator as an endangered species’ was first mentioned in 1979 in the eponymous paper by future NIH director James Wyngaarden [2]. Twenty years later, Leon Rosenberg aptly called physician-scientists as both ‘endangered and essential’ and continued to identify some of the underlying challenges and possible solutions [3]. In 2016 the National Institutes of Health (NIH) convened a series of workshops to update programmatic, system-wide solutions to the enduring challenge of clinician-investigator recruitment and retention [4]. The percentage of US physicians engaged in patient-oriented research has steadily declined for the past 30 years, from a peak 4.7% in the 1980s to 1.5% as of 2012 [5, 6]. A variety of reasons have been posited for this decline, including: an increasing portion of students with a large academic debt, an increase in the amount of time required to prepare for a research career; and the perception by physicians that they may not be competitive with PhDs.

For psychiatry, the decreasing number of physician-scientists is especially problematic. Viewed in light of challenges such as the public health costs of mental illness, addiction and an aging population, as well as opportunities to utilize scientific advances to improve prevention, early intervention and treatment of psychiatric disorders, the need for psychiatrist-researchers is particularly urgent. In addition to the common concerns about personal economic disincentives and long duration of training, medical students and psychiatry residents face limitations in the availability of appropriate research education and training opportunities.

The need in the area of child mental health is particularly great given the shortage of child and adolescent psychiatrists (CAPs), the prevalence and diversity of child and adolescent mental disorders, their public health costs, and their potential long-term impact on society. One quarter of the U.S. population is under the age of 18, and at least 13% of these children and adolescents (some 15 million individuals) have diagnosable psychiatric disorders [7–9]. Nine to 13% of U.S. children and adolescents meet the definition of a ‘serious emotional disturbance’ [10]. However, in 2017, only about one in nine emotionally disturbed U.S. children and adolescents received any mental health services [11]. It is estimated that there is only one CAP per 1807 children and adolescents who are in need of mental healthcare [12]. As of 2016, no state in the US has what professional groups would deem a sufficient number of CAPs to serve children in need [12].

Mental illness in childhood is costly and a burden to society, especially when the costs associated with human services, educational interventions and juvenile justice interventions are added to those of psychiatric and mental health services. For example, the estimated 1998 annual expenditures for mental health services (specialty mental health and general health sectors) was $11.8 billion, or about $173 per child. This is nearly a threefold increase from the 1986 estimate of $3.5 billion (not accounting for inflation). However, a recent analysis that encompassed youth overall (ages 0–24) and not just mental health service costs, but also health, productivity, and crime costs associated with mental illness in youth, estimated the annual expenditures in 2007 at closer to 247 billion [13]. Although early diagnosis and treatment can help to defray societal costs of mental illness among children, many go undiagnosed [14] due to lack of access to mental healthcare services, and instead end up in the juvenile justice system [15]. Of the 2 million children arrested each year, an estimated 50–75% have a mental health disorder [16].

More physician-scientists are needed to pursue research careers to understand the pathogenesis, treatment, and prevention of this costly set of disorders. Future progress depends on the recruitment, training and support of: (1) CAPs, pediatricians, and psychologists who are conversant with advances in genetics and the clinical neurosciences; and (2) basic scientists in the neurosciences and human genetics who are familiar with the phenomenology of childhood-onset neuropsychiatric disorders and who can utilize this knowledge as they approach potentially relevant basic science problems. Research educational programs are needed that can introduce scientific advances and prepare physician-scientists for interdisciplinary careers through the acquisition of advanced degrees and working collaborations with scientists in related fields.

The traditional model of training in psychiatry provides only limited opportunities for medical students and residents to participate in child and adolescent clinical services. That approach also does not typically encourage trainees to pursue formal training in research. As a result, promising medical students who have both a passion for research as well as a commitment to the wellbeing of children and adolescents have few opportunities to pursue these goals immediately following their graduation from medical school. There certainly have been laudable and varied approaches to enhance research literacy and training during psychiatric residency [17–20] and CAP fellowship [21], but it is not clear that such interventions have had a long-term impact in the number of independently funded researchers, and particularly in the clinician-scientist tradition. There are inadequate numbers of medical students pursuing specialty careers in academic psychiatry; there is insufficient mentoring at all career levels; and there are far too few institutional and departmental resources devoted to this enterprise. In fact, only about 25% of medical students have a clinical experience in CAP during their psychiatry rotation, leaving them largely unaware of the field and resulting in a large gap in the recruitment and education of future child and adolescent psychiatrists overall [22], including CAP physician-scientists. Although the majority of medical schools have CAP electives, fewer than 5% of medical students participate in them and the required medical student didactics in CAP are minimal [22]. Innovative approaches to increase the exposure of CAP during medical school have proven effective in raising awareness about the field [23], including its research opportunities, and in enhancing recruitment into psychiatry [24].

To address these educational challenges in psychiatric residency, we crafted, with the assistance of a national task force appointed by the American Academy of Child and Adolescent Psychiatry (AACAP), a model curriculum aimed at providing newly graduated physicians with an integrated program that combines training in child and adult psychiatry with early and ongoing formal and ‘hands on’ training in research. [25, 26] The enrollment of a sixteenth cohort into this program provides an opportune time to evaluate the outcomes of its trainees and graduates.

We hypothesized that graduating medical students enrolled into this integrated training program, when compared to peers who were similarly ranked in our original match lists but ultimately pursued residency programs elsewere, would result in a higher rate of clinician-scientists dedicated to careers in child and adolescent psychiatry, and in higher metrics of academic productivity and scientific independence.

## Methods

### Program description and participants

The Albert J. Solnit Integrated Training Program (AJSP) was implemented at the Yale Child Study Center in 2004. Named after the Center’s third director, the AJSP blends training in pediatrics, psychiatry, child and adolescent psychiatry, and research competencies into a 6-year continuous experience. Participants are recruited into the program after graduation from medical school, and upon completion become board-eligible in both general and child and adolescent psychiatry. The program’s overall structure and specific details are available in a prior publication from our group [25] and in the AJSP website [26].

Funding for the AJSP derives from a range of sources, including traditional graduate medical education training slots through Yale-New Haven Hospital and the Veteran’s Administration Hospital, which combined provide most of the support for four of the 6 years of training. The remaining 2 years, largely devoted to protected research activities, are funded through a combination of: (1) an R25 grant from NIMH (MH077823), now in its 14th year, designed to target the specific research goals of the AJSP; (2) a T32 training grant from NIMH (MH018268), now in its 35th year, which supports training infrastructure across a range of relevant disciplines; and (3) philanthropic support.

We have enrolled two participants each year since the program’s inception (the 2004–2010 cohort). We have to date recruited a sixteenth cohort (2019–2025), which we include in the sample’s description but not in the outcome analyses that follow.

### Control group

We used official results from the National Resident Matching Program (‘The Match’, nrmp.org). We obtained the names of all applicants ranked into the AJSP match list for the sixteen cohorts matriculating between 2004 and 2019 (n = 226; median per cohort = 14). Two applicants per cohort matched into the Program each year, and these individuals became our AJSP group. For each AJSP index recruit, we identified two control subjects, who were ranked into the AJSP match list but ultimately went to other programs across the country. Controls were selected based on their nearest rank order in that year’s AJSP match list, and generally within ± 2 slots of each index recruit. We excluded from analysis twelve graduating medical students who matched into psychiatry at Yale, but not specifically into the AJSP, substituting them with the next closest ranked participant in the match list. Given programmatic areas of training overlap between the traditional and AJSP programs at Yale, we considered that comparisons between the two could have biased results to the nil and obscured the unique elements of the AJSP.

### Ethics approval

We obtained approval from the Yale University Institutional Review Board (#2000026883). As a secondary analysis of publicly available data, our study was deemed exempt and did not require informed consent. We provide all of our results in the aggregate and without any potentially identifying personal information.

### Outcomes measures

We first collected information on two proxies of professional identity and commitment to the field of CAP:

#### AACAP membership and boards certification

We extracted membership information from the American Academy of Child and Adolescent Psychiatry website (aacap.org), and boards confirmation from the verifyCert website (abpn.com/verifycert) of the American Board of Psychiatry and Neurology (ABPN). For each participant, we collected board certification (if any) and year of initial certification for both general and child and adolescent psychiatry.

Next, we considered as milestones of research productivity and independence three broad outcomes obtained from publicly available databases:

#### AACAP research awards

We obtained information on three broad categories of competitive research awards conferred each year by AACAP: a) Travel awards to attend its Annual Meeting; b) Pilot research awards ($15,000); and c) Junior investigator awards ($30,000). We counted the number and type of awards received by each participant, as well as the time from program matriculation to receipt of first award.

#### Peer reviewed publications

We collected bibliographic information from the US National Library of Medicine using PubMed (pubmed.gov), and citation metrics from the Data Citation Index of the Web of Science (Clarivate Analytics, webofknowlledge.com). For each participant we determined: (a) Number of publications, starting 1 year after program matriculation; (b) Number of first-authored publications; (c) h-index, defined as the largest number of articles cited at least h times [27]; (d) Cumulative citations; and (e) Mean and highest impact factor (IF) of the journals cited. We then calculated time from program matriculation to first published article.

#### Federal grant funding

We obtained information on federal grant funding using the National Institute of Health’s Research Portfolio Online Reporting Tools (RePORT, report.nih.gov). For each participant we determined: (a) Receipt of K-series award; (b) Receipt of R-series award; and (c) Total dollar amount over funded period. Finally, we calculated time from program matriculation to first conferred award.

### Statistical analysis

We compared variables of interest across AJSP and control groups using Chi square tests for nominal variables, and independent sample t-tests for continuous measures. We used non-parametric tests for two positively skewed variables that were not normally distributed: (a) Rank order in the match list; and (b) Number of publications. In both instances we used the independent samples median test with Yates correction. For time-to-outcome analyses we used the non-parametric Kaplan–Meier estimator. We plotted its results as the 1—cumulative survival function over time and compared the mean times to event using the log rank (Mantel-Cox) test for equality of survival distributions. We conducted all analyses using SPSS 25.0 (Armonk, NY).

## Results

Thirty participants matriculated into the AJSP in the 15 years between 2004 and 2018. They were comparable in rank order to sixty participants who ultimately matched into other accredited programs in the US. Median and interquartile range [IQR] rank order was not different between the AJSP (3 [4]) and control groups (5 [3]; t = 2.1, df = 1, ns).

Demographic and other descriptive characteristics of the AJSP group (including a sixteenth cohort that matriculated in 2019) are summarized in Table 1. Sex distribution has been balanced among the 32 participants, eight of whom (25%) are members of under-represented minorities (URM; four African American, three Latinx, one Native American). One-third of participants have an advanced degree, separate from their MD (16 PhD, 4 MPH or equivalent). Of those degrees, 15 had been conferred prior to matriculation, and 5 were earned as part of academic work during the course of the AJSP. One half of eligible participants received financial support in the form loan relief through the NIH Pediatric Research Loan Repayment Program. Because we had no comparable information on these variables for the control group, we did not make intergroup comparisons.

**Table 1:** Albert J. Solnit Integrated Training Program (AJSP) Participants

	n	%
Unique participants (enrolled 2004–2019)	32	100
Currently Enrolled (2020)	12	38
Graduates (2010–2019)	20	63
Demographic characteristics		
Women	17	53
African American	4	13
Latinx	3	9
Native American	1	3
Advanced degrees	20	63
Upon matriculation	15	47
Upon graduation	5	16
NIH Pediatric Research Loan Repayment Program (among 24 eligible)	12	50
Academic and tenure-earning positions (among 20 graduates)	15	75

Table 2 summarizes our a priori defined outcomes between the AJSP and control groups for the first 15 cohorts. With respect to professional affiliation, AACAP membership was higher in the AJSP than in the control group (97 vs 32%, p < 0.001). General psychiatry board certification among eligible participants was the same between groups (83%), but CAP certification was higher among AJSP participants (85 vs 53%, p < 0.05). Times to board certification did not differ between groups (4.8 ± 1.3 years for general psychiatry, and 6.6 ± 3.6 years for CAP).

**Table 2:** Professional affiliation and research productivity outcomes between AJSP and control groups

	AJSP n = 30		Control n = 60		Statistic
Professional affiliation					
AACAP membership: n (%)	29	97	19	32	x^2^_df=1_ 33.95***
ABPN certification					
Psychiatry: n/eligible (%)	20/24	83	40/48	83	x^2^_df=1_ 0.0
CAP: n/eligible (%)	17/20	85	21/40	53	x^2^_df=1_ 6.06*
AACAP research awards					
Awards					
Any: n (%)	22	73	12	20	x^2^_df=1_ 24.20***
Travel to annual meeting: n (%)	15	50	10	17	x^2^_df=1_ 11.08**
Pilot research: n (%)	17	57	4	7	x^2^_df=1_ 27.95***
Junior investigator: n/eligible (%)	6/20	30	0/40	0	FET***
Number: mean (SD)	2.1	0.8	1.4	0.7	t _df=1_ 2.08*
Peer-reviewed publications					
Any: n (%)	27	90	45	75	x^2^_df=1_ 2.81
Number: mean (SD)	14.1	27.4	6.7	16.5	t _df=1_ 4.07*^a^
Median (IQR)	5.5	16.0	2.0	7.0	
First authored: mean (SD)	4.9	6.4	2.4	5.1	t _df=88_ 2.03*
h index: mean (SD)	9.0	10.0	5.0	5.8	t _df=86_ 2.39*
Cumulative citations: mean (SD)	527	1303	192	470	t _df=86_ 1.76
Impact factor: mean (SD)	6.9	7.8	5.5	5.1	t _df=69_ 0.33
Highest: mean (SD)	15.0	16.0	13.6	17.0	t _df=69_ 0.98
NIH grant funding					
K series: n/eligible (%)	7/20	35	5/40	13	x^2^_df=1_ 4.22*
R series: n/eligible (%)	3/20	15	2/40	5	x^2^_df=1_ 1.75
R series (CAP-related): n/eligible (%)	3/20	15	0/40	0	FET*
Dollar amount (×100,000): mean (SD)	9.3	6.7	9.1	7.4	t _df=10_ 0.05

*AJSP* Albert J. Solnit Integrated Training Program, *IQR* interquartile range, *FET* Fisher’s Exact Test, *AACAP* American Academy of Child and Adolescent Psychiatry, *ABPN* American Board of Psychiatry and Neurology

^a^Independent samples median test with Yates correction

* p < 0.05 **p < 0.01 ***p < 0.001

AJSP graduates and trainees outperformed their control group peers in most research outcomes: First, receipt of AACAP research awards was higher in the AJSP than in the control group (73 vs 20%, p < 0.001). All three types of research awards were more common in the AJSP group (p ≤ 0.001 for each). The number of awards per recipient was higher among the AJSP participants (2.1 ± 0.8 vs 1.4 ± 0.7, p = 0.04). Next, AJSP participants had more publications in PubMed than control group participants (14.1 ± 27.4 vs 6.7 ± 16.5, p = 0.044), more first-authored publications (4.9 ± 6.4 vs 2.4 ± 5.1, p = 0.046), and a higher h-index (9.0 ± 10.0 vs. 5.0 ± 5.8, p = 0.019). Cumulative citations and impact factor metrics did not differ across groups. Finally, NIH K- or R-series grant funding success rate among graduates was higher among AJSP than control group participants (35 vs 13%, p = 0.04). Neither the number of years awarded, nor the dollar amounts funded differed statistically between the groups. Of note, of the five R-series grants awarded, the three in the AJSP group were on topics related to CAP or developmental science; the two in the control group were not (p = 0.04).

**Fig. 1:**
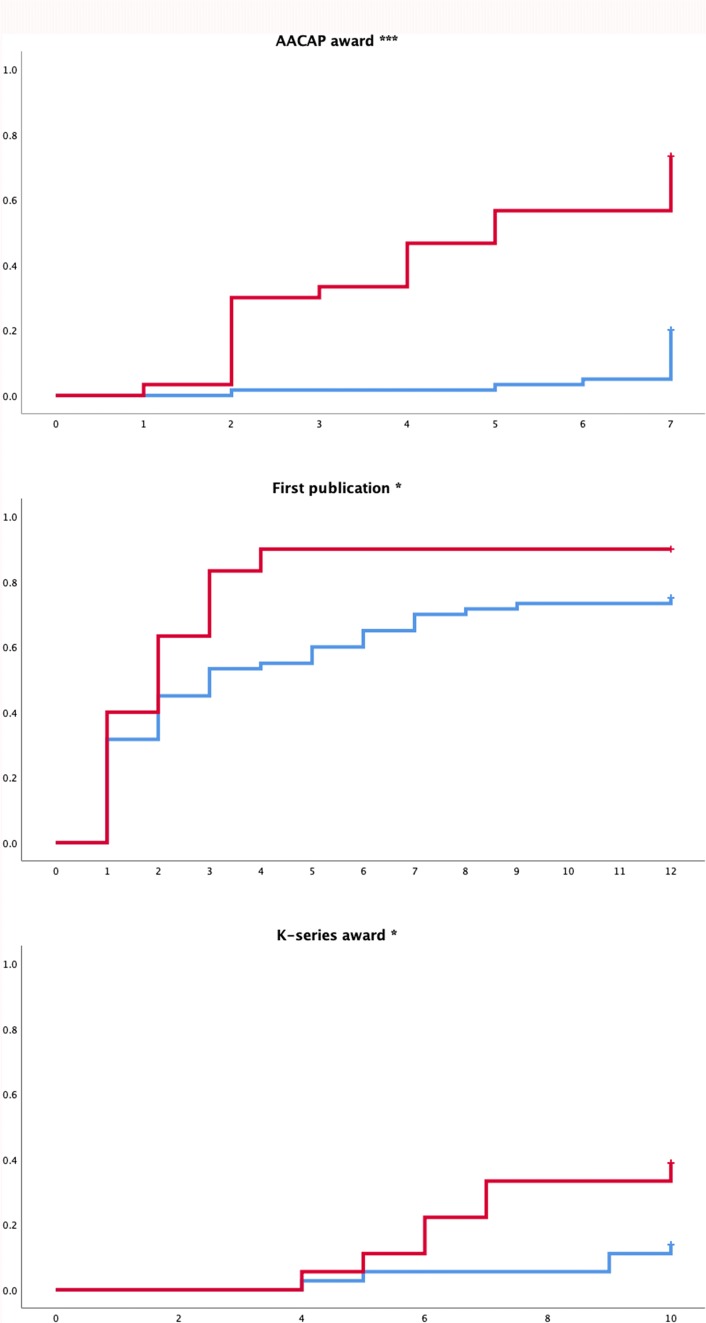
Years to first of three outcomes of interest between AJSP and control groups.  : AJSP (Albert J. Solnit Integrated Training Program),  :Control group. x axes denote time (in years); y axes denote fraction of subjects with outcome of interest, calculated as 1—the survival function obtained though the Kaplan–Meier estimator. p values calculated with log rank (Mantel-Cox) tests for equality of survival distributions. *p < 0.05, ***p < 0.001

Figure 1 depicts visually the time-to-first of three outcomes of interest between the AJSP and control groups. Years to first AACAP award was shorter in the AJSP than in the control group (4.7 ± 0.4 vs 6.9 ± 0.9; log rank = 35.05, df = 1, p < 0.001). Years to first peer-reviewed publication was likewise shorter (2.9 ± 0.6 vs 5.3 ± 0.6; log rank = 5.44, df = 1, p = 0.02), as was time to first K-series award (8.6 ± 0.5 vs 9.6 ± 0.2; log rank = 4.66, df = 1, p = 0.03).

## Discussion

AJSP graduates and trainees outperformed their control group peers in most outcome measures of interest:

We incorporated membership as a proxy variable to determine trainees’ commitment to careers in child and adolescent psychiatry, particularly after graduation. There is no other streamlined way to determine career trajectory. The three-fold higher AACAP membership rate in the AJSP group indicates its participants are and remain committed to working in the field. This is no trivial point, given that many promising would-be investigators in child psychiatry end up doing research in other fields given the long training duration and lag time necessary for subspecialized work with children and adolescents. Our findings suggest, albeit indirectly, that graduates of the AJSP are more likely to become active contributors not just to research in psychiatry, but specifically within the area of child and adolescent psychiatry. The high success rate in applying for AACAP awards, the larger number of awards received, and the shorter duration to first award all suggest that the AJSP has been effective in ‘socializing’ its participants from early on in their training into the work of, and opportunities provided by the Academy.

We tracked ABPN certification as a means of determining trainee’s clinical competency. AJSP participants were more likely to get board-certified in CAP compared to control peers, and despite the emphasis on research in the residency program, AJSP trainees did not take significantly longer to receive their initial board certifications compared to their control group peers. It should be noted that board certification may not be pursued by some graduates—particularly those opting for translational research careers, or those going to academic positions in which there may be less time pressure to obtain certification. Despite these caveats, AJSP graduates were still more likely to become board certified in CAP.

The majority of participants in both groups published peer-reviewed work indexed by the National Library of Medicine. However, participants in the AJSP were considerably more prolific, as reflected by a greater number of published and first-authored articles, and a shorter time to first publication. The fact that mean and highest impact factor did not differ between the groups indicates that participants in the AJSP were not simply publishing more articles, but publishing in comparable quality venues. The h-index of AJSP participants was higher, suggesting more heavily cited and visible scientific output. This finding is notable in that the h-index, like the impact factor, has shortcomings that include the time required to accrue citations [28]. Indeed, cumulative citations did not differ between the two groups, showing the long lag time needed for citation accrual.

A commonly used ultimate metric of scientific independence is receipt of NIH funding, particularly R-series grants. Participants in the AJSP had higher success rates in obtaining K-, but not R-series funding. The average dollar amount did not differ between groups. Given the low number of R-series awards (five) among this sample of 90 participants, and the long lag time to obtain a first R (an average of 10.6 ± 1.5 years from program enrollment) it behooves educators and funding agencies contemplating disbursement of funds to consider alternative metrics of scientific productivity in the shorter term.

### Active components of the AJSP

We would have been delighted to take into the AJSP any one of the applicants in our overall sample of ninety. Indeed, the sixty applicants who did not come to Yale were just as highly ranked (by us) as those who stayed. Each and every one of them was an exceptional individual with an impressive track record even that early in their medical education. Given this comparability in applicant qualifications, and given that many of the programs they went on to have several commonalities with our program (such as resources, grants, and infrastructure), we conclude that the differences in outcomes we found are attributable to the unique characteristics of the AJSP. We go on to outline what we consider to be these ‘active ingredients’.

In developing and refining the AJSP iteratively over its first 15 years in operation, we have followed a dozen guiding principles we consider integral to its success:

1. *Early identity formation.* Trainees incorporate direct experience in caring for children and families as they develop as both clinicians and independent physician-scientists;
2. *Mentorship and career development.* Trainees are assigned a research faculty mentor from their first year onwards. Mentors are accomplished investigators with a sustained record of competitive research funding and active research programs. Mentors have a major responsibility for supervising the trainee, providing assessment and constructive feedback, documenting the trainee’s research progress and performance, and assisting with career development and application for a K award. Participants work closely with their research mentor and residency training director to tailor an appropriate sequence of clinical training and research education and experience;
3. *Integrative program structure.* The program integrates research with clinical training, and child and adolescent clinical training with adult training by structuring these experiences concurrently and using shared group learning and faculty supervision to foster integration. Unlike traditional training models in psychiatry, both research and child psychiatry training begin early and continue throughout the residency. AJSP trainees are also encouraged (but not required) to have clinical mentors that guide them to develop particular clinical skills of interest across the training period;
4. *Optimal focus on child psychiatry*. Wherever possible, child psychiatry rotations are substituted for ones in adult psychiatry, if/as permitted by ACGME and ABPN requirements for both adult and child and adolescent psychiatry. For example, pediatric medicine is scheduled as the required primary care medicine rotation;
5. *Foundation of core clinical training*. The AJSP provides a full range of inpatient and outpatient experiences that support the acquisition of fundamental clinical skills in adult and child psychiatry. Residents achieve competencies in all six areas identified by the ACGME: medical knowledge, patient care, practice-based learning, interpersonal and communication skills, systems-based practice, and professionalism. They are clinically evaluated using the Milestones of the ABPN. Their strong clinical foundation serves as the basis for evidence-based clinical practice and the development of advanced research skills toward a career as independent investigators;
6. *Evidence*-*based perspective.* The principles and practice of Evidence-Based Medicine (EBM) anchor the curriculum and training experiences in both adult and child psychiatry. Regularly scheduled EBM seminars build skills in evidence-based clinical practice;
7. *Early research immersion.* Intensive immersion in clinical psychiatry research starting during the second year fosters early professional identity development as an investigator and is expected to reduce attrition from long-term commitment to research careers;
8. *Formal research training.* Optimally, training in the AJSP includes coursework leading to a Ph.D. or master’s degree, if such a degree was not already acquired and/or as determined by a trainee’s learning needs assessment. Concurrent formal research training that is separately supported is available to AJSP trainees through the Investigative Medicine Program or the Yale Department of Epidemiology and Public Health. This formal training can begin as early as the third year;
9. *Instruction in responsible conduct of research (RCR).* A robust approach to RCR includes formal educational activities supplemented by lectures, workshops and substantial face-to-face discussions. In addition, trainees become familiar with policies and procedures addressing academic misconduct, conflict of interest and conflict of commitment, human subject research protection, and (when relevant), institutional animal care and use;
10. *Comprehensive research experience*. The AJSP provides a research experience that is comprehensive in terms of time, formal curriculum, mentorship, structured evaluation and feedback. These components are essential for professional growth and development. Over the course of training, AJSP trainees are guided through progressive, supervised research experiences, from critical appraisal of the literature, literature reviews and secondary data analyses, through increasingly complex research projects, independent study design and grant-writing, culminating in the submission of an application for a career development (K-series) award in their final year;
11. *Commitment to the enhanced recruitment of under*-*represented minorities (URMs).* The term URM refers to those ethnic or racial groups that are underrepresented in the field of medicine, including African American/African, Hispanic/Latinx, and Native American or Pacific Islander. Eight out of 32 (25%) of AJSP trainees identify as URMs. This fraction is considerably higher than 9%, the AAMC’s reported national average of URM physicians practicing medicine [29]. Multiple reports have also highlighted the severe need for more physician-scientists with URM backgrounds, with only 7% of NIH grant awardees being URMs [30]. In this context, recruitment of URMs with the intent of improving diversity in the physician and the CAP physician-scientist workforce has been an important programmatic goal; and
12. *Debt repayment.* Scheduled research time of at least 80% in the final 2 years of the AJSP qualifies trainees for the NIH Loan Repayment Program. Thus far, one half of eligible residents enrolled in the AJSP have been able to secure debt loan repayment through this mechanism.

## Limitations

Our study has inherent limitations, beginning with its focus on a single program, which may limit generalizability, particularly to settings with a smaller child psychiatry presence or more limited research infrastructure. In addition, our sample was relatively small, and a 15-year window is not sufficient to assess longer-term outcomes, like those pertaining to NIH R-series funding. We were not able to collect information on self-reported ethnicity or on current academic or tenure-earning positions for all members of the control group, which limited our ability to determine how our participants and graduates fared comparatively. Finally, we were not able to get individual-level data regarding federal debt relief for control group participants, as the LRP dashboard (dashboard.lrp.nih.gov) provides only state-aggregated information.

## Conclusions

In summary, we found that a program specifically designed with the aim of providing specialized training for physician-scientists committed to careers in child and adolescent psychiatry has been able to meet its aims 15 years since inception. Critical to the program’s fiscal wellbeing are two federal grants that combined provide approximately one quarter of its overall funding (17% and 8% through the R25 and T32 mechanisms, respectively). At a time of uncertainty regarding NIH funding, we consider our findings informative in setting priorities and confirming a solid return on investment. Specifically, the 2.8 million dollars in R25 funding since 2004 has already yielded 4.6 million dollars in new grant funding. Aside from the fiscal bottom line, the AJSP has led to the formation of a unique group of clinician-scientists with remarkable scientific creativity, innovation and output. We look forward to continuing to refine the program and are committed to its longevity. We are hopeful that other programs may consider replicating or expanding the AJSP approach, something that the Universities of Colorado and Vermont have already started doing. There is a pressing public health need for these and other innovative approaches to enhance the training of clinician-scientists devoted to ease the suffering of children and adolescents with psychiatric illnesses.

## Data Availability

Not applicable.
